# Treatment of Ganglion Cysts

**DOI:** 10.1155/2013/940615

**Published:** 2013-05-28

**Authors:** Matthew Suen, B. Fung, C. P. Lung

**Affiliations:** Department of Orthopaedics and Traumatology, University of Hong Kong Medical Centre, Queen Mary Hospital, Pokfulam Road, Hong Kong

## Abstract

Ganglion cysts are soft tissue swellings occurring most commonly in the hand or wrist. Apart from swelling, most cysts are asymptomatic. Other symptoms include pain, weakness, or paraesthesia. The two main concerns patients have are the cosmetic appearance of the cysts and the fear of future malignant growth. It has been shown that 58% of cysts will resolve spontaneously over time. Treatment can be either conservative or through surgical excision. This review concluded that nonsurgical treatment is largely ineffective in treating ganglion cysts. However, it advised to patients who do not surgical treatment but would like symptomatic relief. Compared to surgery, which has a lower recurrence rate but have a higher complication rate with longer recovery period. It has been shown that surgical interventions do not provide better symptomatic relief compared to conservative treatment. If symptomatic relief is the patient's primary concern, a conservative approach is preferred, whilst surgical intervention will decrease the likelihood of recurrence.

## 1. Introduction

Ganglion cyst is the most common soft tissue swelling in hand and wrist. It occurs most commonly on the dorsal side of the wrist (70%), followed by volar side (20%) of wrist and tendon sheath of fingers. Most of the ganglion cysts are asymptomatic besides swelling. Most patients sought advice and treatment because of the cosmetic appearance or they were concerned that their ganglion was a malignant growth [1]. Treatment options include reassurance, nonsurgical means like aspiration with or without steroid injections or hyaluronidase and surgical excision. We review the treatment outcome of ganglion in the literature and compare their recurrence and complication rates.

## 2. Methods

Electronic databases of Medline, PubMed, and the Cochrane libraries were searched with the key words “ganglion,” “conservative treatment,” “surgery” and “outcomes.” The inclusion criteria were (1) publication in English and (2) articles concerning the treatment of ganglion of hand and wrist. Recurrence rate, complications, and functional outcome were reviewed. References in review articles were screened for potentially relevant studies not yet identified.

## 3. Reassurance

Majority of patients with ganglion do not have symptoms besides swelling, while others may present with pain, weakness, or paresthesia. Barnes et al. reported in their review that only 19.5% had symptoms other than a mass [2]. Westbrook et al. also reported majority of patients sought advice and treatment because of the cosmetic appearance or they were concerned that their ganglion was a malignant growth, while only 26% consulted because of pain and 8% consulted altered sensation or restricted hand function [1].

Many may not opt for any treatment if they are reassured of the benign nature of the disease. Also, even for painful ganglions, they cause less pain compared to other common orthopaedic problems, like carpal tunnel syndrome and osteoarthritis, in terms of Mean Visual Analogue Pain Scores [3].

The spontaneous resolution rate of untreated ganglion ranged 40–58% (Table 1) [4–7]. Therefore reassurance can be the option if the patients do not want any intervention.

## 4. Conservative Treatment

### 4.1. Aspiration

Aspiration alone is one of the simplest ways to treat ganglion. However, it has high recurrence rates. Most of the studies showed more than half of ganglion treated with aspiration alone will recur (Table 2) [6–25]. Many methods have been tried in order to increase the efficacy. Zubowicz and Ishii reported a recurrence rate of 15% by repeated aspiration up to three times. However, they also noticed the successful rate decreased with those who needed repeated aspiration [10]. Multiple puncture of ganglion wall has not shown to improve the result of simple ganglion aspiration [12].

### 4.2. Steroid

Becker suggested the use of steroid injection in treating ganglion, with 87% resolution rate, based on the initial theory that chronic inflammatory may take part in the pathogenesis of ganglion. Subsequent studies showed variable successful rate. Varley et al. conducted a randomized controlled trial to aspiration with or without steroid and concluded that additional injection of steroid is of no benefit and subcutaneous fat atrophy and skin depigmentation can be the potential complications [11].

### 4.3. Sclerotherapy

Sclerotherapy has been proposed to treat ganglion. Sclerosant was injected into ganglion sac to damage the intimal lining and cause fibrosis to reduce the recurrence rate. Initial study showed high successful rate ranging 78–100%. Mackie et al., however, confirmed ganglion had no intimal lining by histological studies and reported a failure rate as high as 94%. Since there is communication between ganglion and synovial joint, sclerosant might pass from ganglion to the joint and tendon and cause damage to them [16]. Since the publication of these reports, the use of sclerotherapy had declined. New technique had been developed with the aim of causing ganglion sclerosis without the risk of damage to the joints. Gümüş used electrocautery to cause ganglion sclerosis and showed favorite results. This technique had not been widely adopted [18].

### 4.4. Hyaluronidase

The content of ganglion may be too vigorous to be drawn, and thus aspiration may not be complete. Some advocated the use of hyaluronidase, which depolymerizes the hyaluronic acid present in ganglion content. Otu reported a 95% cure rate after a follow-up period of 6 months [19]. Paul and Sochart also showed that the use of hyaluronidase in conjunction with steroid has resulted in significantly higher resolution rate compared to the use of steroid alone, but only 49% of their patients treated by hyaluronidase and steroid had complete resolution, compared to 20% in those treated with steroid [15]. Akkerhuis et al., however, reported a recurrence rate of 77%, for treatment of ganglion with hyaluronidase [20]. Thus, the successful rate had been variable, and hyaluronidase may cause allergic reaction.

### 4.5. Immobilization

Immobilization following aspiration had showed conflicting results. Richman et al. showed that 3-week immobilization after aspiration and multiple puncture had a significantly higher successful rate for dorsal carpal ganglion, but the result for palmar ganglion was inconclusive [21]. On the other hand, Korman et al. concluded that immobilization did not significantly improve the successful treatment of ganglions over perforation and aspiration alone and had the potential adverse effects of inconvenience, economic repercussions, and stiffness [22].

### 4.6. Threat Technique

Gang and Makhlouf introduce the thread technique, by which two sutures were passed through the ganglion at right angles to each other, and each was tied in a loop. The contents of ganglion were expelled by massage at interval. They reported a recurrence rate of 4.8%. However, 11% of the patients had positive culture swabs [24]. Singhal et al. described a similar technique, but the complete resolution rate was only 50% [25].

Taking into account that nearly half of the ganglion would resolve spontaneously, with such a high failure rate, nonsurgical treatment of ganglion was generally ineffective. However, the complications were considered less (Table 3) [6–25]. Some reported zero percent of complication rates, while others reported minor complications like transient pain and swelling. Therefore, nonsurgical treatment can be considered to be an alternative way for symptomatic relief if the patients do not want surgery.

Another advantage of conservative treatment is that aspiration of ganglion contents confirms a benign diagnosis and allays the patients' fear and desire for further treatment. 

## 5. Surgery

In 1976, Angelides and Wallace [26] introduced the techniques of excising the whole ganglion including the cyst, its attachments to the scapholunate ligament, and the involved segment of joint capsule, to reduce the recurrence rate. It is now considered to be the most effective technique.

### 5.1. Recurrence

According to the study conducted by Angelides and Wallace, the recurrence rate can be as low as 1%. However, subsequent recurrence rate of surgical excision reported by the literatures was variable (Table 4) [6, 7, 13, 20, 23, 26–29, 31–47], with the range of 0–31.2%. There were only two randomized controlled trials comparing the recurrence rate of conservative treatment to surgery. Limpaphayom and Wilairatana compared aspiration, steroid injection, and immobilization with surgery, while Akkerhuis et al. compared hyaluronidase with surgery. Both of them reported surgery had a lower recurrence rate [20, 23].

### 5.2. Complications

Complications for surgical excision included wound infection, neuroma formation, hypertrophic scar, median nerve, and radial artery damage, with complication rate ranging 0–56% (Table 5) [6, 7, 13, 20, 23, 26–29, 31–47]. In Dias and Buch's cohort study, surgery (20%) had a higher complication rate compared with aspiration (5%) or reassurance [6].

Scapholunate instability has been reported after dorsal wrist ganglion excision. Some suggested periscaphoid ligamentous injury was a cause of ganglion rather than a complication of surgery [30, 49]. Kivett et al. examined 61 postganglionectomy patients by physical examination and radiography and concluded that ganglion excision did not de-stabilise the wrist [50].

### 5.3. Mobility and Other Outcomes

Surgery may not result in favourable outcomes. Angelides et al. reported 1.2% of patients had 0–10 degree loss of volar flexion after surgery, although this had no functional significant [26]. Sanders studied nine patients with occult dorsal ganglion. One out of eight who attended followup had residual pain after surgery, while three out of eight had limited motion [48]. Clay and Clement reported that while surgery resulted in improvement of pain in 79%, it worsen the pain in 8% of patients. 17% of patients complained of weakened grip with 2% demonstrating loss of grip strength of more than 20% compared with opposite hand [28]. Residual pain, limited range of motion, and weaken grip were also reported in other studies (Table 6).

Dias conducted two prospective cohort comparing the outcomes of dorsal and palmar ganglions, respectively, treated by surgery with those treated by reassurance and aspiration. No significant difference was found in persistent symptoms and symptom relief among three groups. However, those treated with surgery had significantly higher recovery times, with averaged 14.1 days and 10.9 days off work for palmar and dorsal wrist ganglion excision, respectively, compared to averaged 3.5 days and 3.2 days for aspiration of palmar and dorsal wrist ganglion [5, 6] (Table 7).

### 5.4. Arthroscopic Excision

In 1995, Osterman and Raphael described a technique of arthroscopic excision of dorsal wrist ganglia. Arthroscopic resection has the potential advantages of minimizing the surgical scar and permits evaluation of any intra-articular pathologic condition of either midcarpal or radiocarpal joints [38]. 

Majority of initial reports on recurrence rate look more favourable than open excision (Tables 4 and 5). However, a prospective, randomized study in 2008 showed rates of recurrence with arthroscopic dorsal ganglion excision (3/28) are comparable with and not superior to those of open excision (2/23). Additional long-term comparative studies are needed to accurately differentiate the efficacy of open and arthroscopic techniques [36].

## 6. Conclusion

Majority of patients with ganglion do not have symptoms. Given that the spontaneous resolution rate of ganglion can be as high as 58%. Reassurance and observation can be the option if the patients are asymptomatic or do not want any intervention. Nonsurgical treatments of ganglion including aspiration, steroid injection sclerotherapy, and hyaluronidase were generally ineffective. However, since they had lower complication rates, they can be used for symptomatic relief if the patients do not want surgery. Surgery had a lower recurrence rate than conservative treatment. However it has higher rates of complication and longer recovery period, and the rate of symptomatic relief may not be higher than other treatments.

## Figures and Tables

**Table 1:** 

	FU	Resolution rate
Carp and Stout 1928	3 years	7/12 (58%)
McEvedy 1962 [4]	10 years	10/21 (48%)
Zachariae and Vibe-Hansen 1973 [5]	6 years	40/101 (40%)
Dias and Buch 2003 [6]	63 months	20/38 (53%)
Dias et al. 2007 [7]	70 months	23/55 (42%)

**Table 2:** 

	FU	Failure rate*	
Aspiration			

Nield and Evans 1986 [8]	1 year	20/34 (59%)	
Esteban et al. 1986 [9]	27 months (3–71)	6/17 (35%)	
Zubowicz and Ishii 1987 [10]	1 year–20 months	7/47 (15%)	
Varley et al. 1997 [11]	48 months (26–89)	28/42 (67%)	
Stephen et al. 1999 [12]	1 year	35/51 (69%)	

Aspiration with or without steroid			

Dias and Buch 2003 [6]	5 years	18/38 (47%)	
Dias et al. 2007 [7]	70 months	45/78 (58%)	

Steroid			

Wright et al. 1994 [13]	5 years (2–11 years)	20/24 (83%)	
Breidahl and Adler 1996 [14]	12 months	6/10 (60%)	
Paul and Sochart 1997 [15]	2 years	28/35 (80%)	
Varley et al. 1997 [11]	46 months (26–89)	29/43 (67%)	

Sclerotherapy			

Mackie et al. 1984 [16]	3 months	15/16 (94%)	Sclerosant (sodium tetradecyl disulphate)
Dogo et al. 2003 [17]	24–36 months	1/29 (3.4%)	Sclerosant (hypertonic saline)
Gümüş 2009 [18]	17 months (6–29)	1/17 (5.9%)	Transcutaneous electrocauterization

Hyaluronidase			

Otu 1992 [19]	6 months	17/349 (5%)	
Paul and Sochart 1997 [15]	2 years	18/35 (51%)	
Akkerhuis et al. 2002 [20]	1 year	33/43 (77%)	

Aspiration + multiple puncture			

Richman et al. 1987 [21]	22 months	32/45 (71%)	
Korman et al. 1992 [22]	1 year	18/36 (50%)	
Stephen et al. 1999 [12]	1 year	32/41 (78%)	

Aspiration + multiple puncture + immobilization			

Richman et al. 1987 [21]	22 months	24/42 (57%)	
Korman et al. 1992 [22]	1 year	16/33 (48%)	

Aspiration + steroid + immobilization			

Limpaphayom and Wilairatana 2004 [23]	6 months	8/13 (62%)	

Thread technique			

Gang and Makhlouf 1988 [24]	Min. 6 months	3/62 (4.8%)	
Singhal et al. 2005 [25]	2 years	13/26 (50%)	

*Failure rate = recurrence + in complete resolution.

**Table 3:** 

	Method	Complication rate	Complications
Esteban et al. 1986 [9]	Aspiration	0	
Dias and Buch 2003 [6]	Aspiration/steroid	5%	Scar tender
Dias et al. 2007 [7]	Aspiration/steroid	2/78 (2.56%)	
Paul and Sochart 1997 [15]	Steroid	4/35 (11%)	Superficial infection, mild localized rash, and small area of depigmentation
Dogo et al. 2003 [17]	Hypertonic saline	0	50% swelling, 6/29 severe pain
Gümüş 2009 [18]	Transcutaneous electrocauterization	0	8 cases of transient swelling and pain
Paul and Sochart 1997 [15]	Hyaluronidase	1/35 (2.9%)	Superficial infection
Richman et al. 1987 [21]	Multiple puncture	0	
Richman et al. 1987 [21]	Immobilization	0	
Limpaphayom and Wilairatana 2004 [23]	Immobilization	0	
Gang and Makhlouf 1988 [24]	Thread technique	7/62 (11%)	Positive culture swab
Singhal et al. 2005 [25]	Thread technique	3/26 (12%)	Localized rash, mild restriction

**Table 4:** 

		FU	Recurrence rate
Open excision			

Angelides and Wallace 1976 [26]	Dorsal	9 months–25 years	3/346 (0.87%)
Janzon and Niechajev 1981 [27]		5 years	21/144 (15%)
Clay and Clement 1988 [28]	Dorsal	28 months (12–74 months)	2/51 (3.9%)
Watson et al. 1989 [29, 30]	Dorsal and palmar	16 years	0/10 (0%)
Jacobs and Govaers 1990 [31]	Palmar	70 months (3–220)	20/71 (28%)
Wright et al. 1994 [13]	Palmar	5 years (2–11 years)	14/72 (19%)
Filan and Herbert 1996 [32]	Recurrent dorsal	14 months (12–22)	0/7 (0%)
Hwang et al. 1999 [33]	Dorsal		1/19 (5.3%)
Faithfull and Seeto 2000 [34]	Dorsal and palmar	65 months (6–133)	6/59 (10%)
Gündeş et al. 2000 [35]	24 dorsal	27 months (6–48)	8.3%
	16 volar		31.2%
Akkerhuis et al. 2002 [20]	Wrist and foot	12 months	11/46 (24%)
Limpaphayom and Wilairatana 2004 [23]	Dorsal	6 months	2/11 (18%)
Kang et al. 2008 [36]	Dorsal	12 months	2/23 (8.7%)
Rocchi et al. 2008 [37]	Palmar	24 months	1/25 (4%)

Arthroscopy + open			

Dias and Buch 2003 [6]	Palmar	5 years	33/79 (42%)
Dias et al. 2007 [7]	Dorsal	70 months	40/103 (39%)

Arthroscopic resection			

Osterman and Raphael 1995 [38]	Dorsal		0/18 (0%)
Luchetti et al. 2000 [39]	Dorsal	16 months	2/34 (5.9%)
Ho et al. 2001 [40]	Dorsal	25 months (6–44)	5/19 (26%)
	Palmar	16.4 months (10–25)	0/5 (0%)
Nishikawa et al. 2001 [41]	Dorsal	20 months	2/37 (5.4%)
Shih et al. 2002 [42]	Dorsal	26.8 months (15–37)	0/32 (0%)
Rizzo et al. 2004 [43]	Dorsal	47.8 months (28–97)	2/41 (4.9%)
Mathoulin et al. 2004 [44]	Dorsal	34 months (12–46)	4/96 (4.2%)
	Palmar	26 months (12–39)	0/32 (0%)
Rocchi et al. 2006 [45]	Dorsal and palmar	15 months (3–26)	2/47 (4.3%)
Kang et al. 2008 [36]	Dorsal	12 months	2/28 (7.1%)
Rocchi et al. 2008 [37]	Palmar	24 months	3/25 (12%)
Edwards and Johansen 2009 [46]	Dorsal	Min. 24 months	0/55 (0%)
Chen et al. 2010 [47]	Dorsal and palmar	15.3 months	1/15 (6.7%)

**Table 5:** 

		Complication rate	
Open excision			

Angelides and Wallace 1976 [26]	Dorsal	0/346 (0%)	1.2% had 0–10 degree loss of volar flexion
Janzon and Niechajev 1981 [27]		Not reported	
Clay and Clement 1988 [28]	Dorsal	0/51 (0%)	1 had evidence of scapholunate dissociation
Watson et al. 1989 [29, 30]	Dorsal and palmar	Not reported	
Jacobs and Govaers 1990 [31]	Palmar	20/71 (28%)	20 had unsatisfactory scar (2 had evidence of neuroma), 20 had evidence of median nerve damage
Wright et al. 1994 [13]	Palmar	6/72 (8.3)	superficial infection, tendinitis, and pain dystrophy
Filan and Herbert 1996 [32]	Recurrent dorsal	Not reported	
Hwang et al. 1999 [33]	Dorsal	3/19 (16%)	1 suture abscess, 1 loss of wrist flexion of 45 degree, and 1 transient neuropraxia
Faithfull and Seeto 2000 [34]	Dorsal and palmar	Not reported	
Gündeş et al. 2000 [35]	24 dorsal	12.5%	1 had evidence of radial nerve injuries
	16 volar	56%	2 had evidence of median nerve injuries, 2 had radial artery injuries
Akkerhuiset al. 2002 [20]	Wrist and foot	Not reported	
Limpaphayom and Wilairatana 2004 [23]	Dorsal	0/11 (0%)	
Kang et al. 2008 [36]	Dorsal	0/23 (0%)	
Rocchi et al. 2008 [37]	Palmar	7/25 (28%)	4 radial artery injuries, 2 partial stiffness of the wrist, and 1 neuropraxia

Arthroscopy + open			

Dias and Buch 2003 [6]	Palmar	20%	
Dias et al. 2007 [7]	Dorsal	8/103 (7.8%)	3 numbness, 4 scar tender, and 1 keloid

Arthroscopic resection			

Osterman and Raphael 1995 [38]	Dorsal	0/18 (0%)	
Luchetti et al. 2000 [39]	Dorsal	0/34 (0%)	
Ho et al. 2001 [40]	Dorsal	0/19 (0%)	
	Palmar	0/5 (0%)	
Nishikawa et al. 2001 [41]	Dorsal	0/37 (0%)	
Shih et al. 2002 [42]	Dorsal	Not reported	
Rizzo et al. 2004 [43]	Dorsal	10/47 (21%)	10 postoperative stiffness
Mathoulin et al. 2004 [44]	Dorsal	0/96 (0%)	
	Palmar	1/32 (3.1%)	1 hematoma
Rocchi et al. 2006 [45]	Dorsal and palmar	4/47 (8.5%)	1 radial artery injury, 1 haematoma, and 2 axonotmesis
Kang et al. 2008 [36]	Dorsal	1/41 (2.1%)	1 neuropraxia
Rocchi et al. 2008 [37]	Palmar	2/25 (8%)	1 neuropraxia, 1 injury to a branch of the radial artery
Edwards and Johansen 2009 [46]	Dorsal	3/55 (5.5%)	3 extensor tenosynovitis
Chen et al. 2010 [47]	Dorsal and palmar	1/15 (6.7%)	1 case of transient paresthesia

**Table 6:** 

	Residual pain	Limited ROM	Reduced grip power	Loss of function
Sanders 1985 [48]	13%	38%	0	0
Clay and Clement 1988 [28]	Improved in 79%, worsen in 8%	17%	0	0
Wright et al. 1994 [13]	8.3%	2.8%	17%	0
Faithfull and Seeto 2000 [34]	14%	0	0	10%
Dias and Buch 2003 [6]	16%	10%	23%	0
Dias et al. 2007 [7]	27%	15%	34%	0

**Table 7:** 

	Mean recovery time	Time off work
Open		

Janzon and Niechajev 1981 [27]		Majority 10–20 days
Jacobs and Govaers 1990 [31]		Majority 7–18 days (median 2.5 weeks)
Rocchi et al. 2008 [37]	15 days	23 days

Open + arthroscopy		

Dias and Buch 2003 [6]		14.1 days
Dias et al. 2007 [7]		10.9 days

Arthroscopy		

Luchetti et al. 2000 [39]		15 days
Nishikawa et al. 2001 [41]	16 days (7–56)	
Rocchi et al. 2008 [37]	6 days	10 days
